# Investigating the impact of the COVID-19 pandemic on recovery colleges: multi-site qualitative study

**DOI:** 10.1192/bjo.2024.70

**Published:** 2024-05-16

**Authors:** Merly McPhilbin, Katy Stepanian, Caroline Yeo, Daniel Elton, Danielle Dunnett, Helen Jennings, Holly Hunter-Brown, Jason Grant-Rowles, Julie Cooper, Katherine Barrett, Mirza Hamie, Peter Bates, Rebecca McNaughton, Sarah Trickett, Simon Bishop, Simran Takhi, Stella Lawrence, Yasuhiro Kotera, Daniel Hayes, Larry Davidson, Amy Ronaldson, Tesnime Jebara, Cerdic Hall, Lisa Brophy, Jessica Jepps, Sara Meddings, Claire Henderson, Mike Slade, Vanessa Lawrence

**Affiliations:** Institute of Mental Health, School of Health Sciences, University of Nottingham, UK; Department of Health Service and Population Research, Institute of Psychiatry, Psychology, and Neuroscience, King's College London, UK; Buildings, Energy & Environment Research Group, Department of Architecture & Built Environment, University of Nottingham, UK; RECOLLECT Lived Experience Advisory Panel (LEAP), Kings College London, UK; College of Health, Wellbeing and Life Sciences, Sheffield Hallam University, UK; Peter Bates Associates, Nottingham, UK; Nottingham University Business School, University of Nottingham, UK; Research Department of Behavioural Science and Health, Institute of Epidemiology and Health Care, University College London, UK; Department of Mental Health and Addiction Services, Yale School of Medicine, Connecticut, USA; St Pancras Hospital, Camden and Islington NHS Foundation Trust, London, UK; School of Allied Health, Human Services and Sport, La Trobe University, Australia; Duncan Macmillan House, ImROC, Nottingham, UK; Institute of Mental Health, School of Health Sciences, University of Nottingham, UK; and Division of Health and Community Participation, Faculty of Nursing and Health Sciences, Nord University, Norway; Social Epidemiology Research Group, Department of Health Service and Population Research, Institute of Psychiatry, Psychology and Neuroscience, King's College London, UK

**Keywords:** Recovery, recovery college, COVID-19, collaborative data analysis, qualitative research

## Abstract

**Background:**

During the COVID-19 pandemic, mental health problems increased as access to mental health services reduced. Recovery colleges are recovery-focused adult education initiatives delivered by people with professional and lived mental health expertise. Designed to be collaborative and inclusive, they were uniquely positioned to support people experiencing mental health problems during the pandemic. There is limited research exploring the lasting impacts of the pandemic on recovery college operation and delivery to students.

**Aims:**

To ascertain how the COVID-19 pandemic changed recovery college operation in England.

**Method:**

We coproduced a qualitative interview study of recovery college managers across the UK. Academics and co-researchers with lived mental health experience collaborated on conducting interviews and analysing data, using a collaborative thematic framework analysis.

**Results:**

Thirty-one managers participated. Five themes were identified: complex organisational relationships, changed ways of working, navigating the rapid transition to digital delivery, responding to isolation and changes to accessibility. Two key pandemic-related changes to recovery college operation were highlighted: their use as accessible services that relieve pressure on mental health services through hybrid face-to-face and digital course delivery, and the development of digitally delivered courses for individuals with mental health needs.

**Conclusions:**

The pandemic either led to or accelerated developments in recovery college operation, leading to a positioning of recovery colleges as a preventative service with wider accessibility to people with mental health problems, people under the care of forensic mental health services and mental healthcare staff. These benefits are strengthened by relationships with partner organisations and autonomy from statutory healthcare infrastructures.

Personal mental health recovery is a process involving feeling connected and empowered, building hope for the future, personal identity and meaning in life.^1^ Recovery colleges are recovery-focused adult education initiatives, providing courses designed and facilitated by both people with professional and lived mental health expertise.^2^ Recovery colleges bring benefits to self-esteem, sense of identity, hope, social networks, lifestyle, quality of life and goal achievement for those attending for their own mental health needs (referred to hereafter as ‘students’).^3^ Recovery colleges vary in their eligibility criteria, location, course content and approaches to supporting students’ personal goals.^4^ A national survey conducted in 2021 to characterise recovery college variation in England identified 88 recovery colleges operating at a cost of £20 000 000 to the National Health Service (NHS), and attended by approximately 36 000 individuals per year.^5^ Three clusters of recovery colleges were identified: strengths-oriented (NHS Trust-affiliated), community-oriented (not NHS Trust-affiliated and focused on social connectedness) and forensic (NHS Trust-affiliated, with majority male student population). Surveyed recovery college managers reported that their responses were affected by the COVID-19 pandemic, which presented an unprecedented challenge for mental health service provision. Suicidality, depression and anxiety increased in UK residents among those with and without pre-existing mental health conditions during the pandemic.^6^ At the same time, access to clinical mental health services provided by the NHS decreased as providers withdrew services, offered services in a different form and changed the threshold for admission.^7^ Both mental health services and mental health initiatives, such as recovery colleges, transitioned from face-to-face to digital delivery using videoconferencing platforms.^8^ Unlike clinical mental health services, most recovery colleges accept self-referrals and are available for any member of the public to attend.^5^ Digitally delivered recovery college courses were felt to support students to self-regulate their mental health challenges and stress levels during periods of social distancing and reduced access to care.^9^ However, there has been no multi-site exploration of how recovery college operation evolved in the UK during the pandemic.

## Aims

This study aimed to ascertain how the COVID-19 pandemic changed the operation of recovery colleges in England.

## Method

### Study design

The study was conducted as part of Recovery Colleges: Characterisation and Testing (RECOLLECT 2), a 5-year research programme exploring the effectiveness of recovery colleges in England.^10^ The RECOLLECT 2 Lived Experience Advisory Panel (LEAP) comprised individuals based in England with lived experience of working at recovery colleges, attending recovery colleges as students, using and/or caring for those who use mental health services. Nine LEAP members were involved as co-researchers in the data analysis, three of whom also conducted interviews alongside academic researchers with varied backgrounds in psychology, qualitative social science and occupational therapy, and some with disclosed lived mental health experience.

We used a coproduced approach to conduct a qualitative interview study by sharing power, expertise and responsibility to create and deliver research and generate knowledge.^11,12^ Grounded in interpretivism, we sought to understand participants’ perspectives in context.

### Participants

Our previous national survey aimed to identify all recovery colleges in England via web searches, consultation with recovery college experts and networks, snowball sampling and contacting large organisations likely to have a recovery college embedded within them (referred to as ‘host organisations’ hereafter).^5^ The recovery college manager or another senior member of staff at 63 of the 88 identified recovery colleges participated in the survey. All 63 participants were invited by email to be interviewed.

### Materials and procedures

Researchers and co-researchers were offered interview training and shadowing opportunities. The semi-structured interview schedule was coproduced with co-researchers and recovery college managers attending a meeting held by the national recovery college network, ImROC, and is presented in Supplementary Appendix 1 available at https://doi.org/10.1192/bjo.2024.70. Participants were prompted to elaborate on adaptations implemented to sustain recovery college operation during the pandemic and changes to adult education, coproduction, communication, managerial decision-making and diversity of the student population.

All participants (referred to as ‘managers’ hereafter) provided informed consent in written or electronic form. Interviews were conducted via Microsoft Teams for Windows (version 1.4.00.20211) between October 2021 and April 2022. Twenty-eight interviews were conducted by researchers (*n* = 6), of which ten were shadowed by co-researchers, and three were conducted by co-researchers (*n* = 3). Interviewing ceased with confidence that a diverse range of perspectives had been explored and that new interviews were adding few additional insights. Interviews were recorded, transcribed verbatim, pseudonymised and analysed with NVivo 14 (Release 1.6.1) for Windows (Lumivero, Denver, Colorado, USA; see https://lumivero.com/products/nvivo/). Researchers recorded reflections on their relationship to the data in a reflexive log.

### Analysis

We conducted a thematic framework analysis^13^ building on a collaborative data analysis approach.^12^ This iterative approach included inductive development, and deductive application, of the framework. The inclusion of lived experience in health research is internationally endorsed,^14,15^ and can bring enhanced trustworthiness and impact to the study materials and results.^16^

First, researchers (*n* = 6) and co-researchers (*n* = 9) read two transcripts and each identified five observations about the impact of COVID-19 on recovery colleges. Researchers and co-researchers attended four video call meetings to group the observations and create a preliminary framework. The preliminary framework (V1) consisted of six themes relating to the impact of COVID-19 on recovery college operation, and is presented in Supplementary Appendix 2.

A smaller group of researchers (*n* = 4) subsequently applied framework V1 to two transcripts and met to resolve coding discrepancies. More subthemes were added to the framework to improve the specificity and fit of the themes to the data. One researcher (M.M.) deductively applied the framework to 16 transcripts, making iterative adaptations until the framework adequately captured the richness of the data.^17^ Framework V2 consisted of five overarching themes and can be viewed in Supplementary Appendix 3.

An in-person collaborative data analysis session was conducted whereby researchers (*n* = 4), co-researchers and an external expert in patient and public involvement provided feedback on the fit of framework V2 to transcribed quotes and their experiential expertise. International experts (*n* = 23) also provided feedback on framework V2 in an online quarterly RECOLLECT International Advisory Board (IAB) meeting. The IAB was convened before the RECOLLECT programme and comprises clinical and non-clinical researchers with world-leading expertise in developing and/or researching recovery colleges or similar initiatives. Linguistic adaptations were made accordingly to produce framework V3, which can be viewed in Supplementary Appendix 4. Researchers (*n* = 4) deductively applied framework V3 to the remaining transcripts, meeting periodically to resolve coding discrepancies. Linguistic adaptations were made to the framework during the manuscript write-up, which were approved by consulted co-researchers.

Organisational and student characteristics of the participants’ recovery colleges were summarised as means and standard deviations, medians and interquartile ranges, and frequencies.

### Ethics statement

The authors assert that all procedures contributing to this work comply with the ethical standards of the relevant national and institutional committees on human experimentation and with the Helsinki Declaration of 1975, as revised in 2008. All procedures involving human participants were approved by the King's College London Research Ethics Office (reference: MRA-21/22-26274). The RECOLLECT project is registered with the public trials registry ISRCTN (ISRCTN10215637).

## Results

Thirty-one recovery college managers participated, meaning that 35% of recovery college managers in England and 49% of the national survey responders were interviewed. No data were collected on why 32 managers did not attend an interview. Organisational and student characteristics of the participants’ recovery colleges are shown in Table 1. A non-statistical comparison with the national survey sample suggests that rural and private sector recovery colleges were underrepresented by the manager participant sample in this interview study. Otherwise, a similar distribution of recovery college characteristics (e.g. location, main organisational affiliation and cluster) were represented.

**Table 1:** Organisational and student characteristics (*N* = 31)

	Mean ± s.d. or *n* (%)
Length of time in operation (years)	5.8 ± 2.5
Location	
Urban	8 (25.8)
Suburban	4 (12.9)
Rural	–
Mixed	19 (61.3)
Number of students per year (median, IQR)	375 (155–960)
Who is the college for?	
Individuals with lived mental health experience, but not using secondary services	23 (74.2)
Individuals using secondary mental health services	30 (96.8)
Individuals using specialist mental health services	30 (96.8)
Informal carers	28 (90.3)
Mental health workers	26 (83.9)
Other mental health staff	26 (83.9)
General public	19 (61.3)
Estimated proportion (%) of ethnic groups attending the college (*n* = 29) (mean ± s.d.)	
Asian/Asian British	6.2 ± 6.3
Black/Black British	8.2 ± 11.1
Mixed/Mixed British	5.7 ± 6.6
White/White British	74.7 ± 21.8
Other	5.1 ± 5.2
Main organisational affiliation (median, IQR)	
Statutory health service, e.g. NHS Trust	24 (77.4)
Non-governmental organisation	10 (32.3)
Local authority, e.g. council	2 (6.4)
Independent	2 (6.4)
Other health, e.g. private healthcare provider	–
Education provider, e.g. university or college	1 (3.2)
Other	1 (3.2)
Number of colleges reporting funding from:	
Clinical commissioning group	12 (38.7)
NHS Trust	19 (58.1)
Charity	8 (25.8)
Self-funded	–
Independent	–
Other	7 (22.6)
Recovery college cluster	
Cluster 1 (strengths oriented)	22 (66.7)
Cluster 2 (community oriented)	7 (21.2)
Cluster 3 (forensic)	2 (6.1)

IQR, interquartile range; NHS, National Health Service.

The final framework comprised five superordinate themes, shown in Table 2. An extended quote table is presented in Supplementary Appendix 5.

**Table 2:** Superordinate themes and corresponding quotes

Theme	Example quote
Theme 1: complex organisational relationships	‘So, I described our Recovery College in COVID as being a third-party sector provider in an NHS system’ (RC02).
	‘Secondary care services are not still not doing an awful lot of face-to-face in-person stuff, which then means a kind of demand backs up. So, we're getting more asked of us’ (RC18).
	‘There started to be this real goodwill emerging, there already was, but [ … ] it was different before the pandemic. People weren't quite as willing to share like course materials and stuff like that’ (RC21).
Theme 2: changed ways of working	‘It just feels it feels quite relentless through the constant change, constant adapting’ (RC19).
	‘Our provision [ … ] has completely changed’ (RC27).
	‘Because things have been so up and down [ … ] it's been difficult sometimes to plan things’ (RC26).
Theme 3: navigating the rapid transition to digital delivery	‘We had to survive. We had to offer people something [ … ] we were very aware that our students were left with nothing’ (RC03).
	‘It had to be [ … ] quite reactive and quite responsive at the time, we didn't really have that opportunity to set up official processes for it because [ … ] the focus was on getting people online and engaged to some degree as quickly as possible’ (RC15).
Theme 4: responding to loneliness and isolation	‘Our clients, they needed a space where they were meeting people [ … ] a lot of them are vulnerable, they were confined to their rooms [ … ] we needed to have a platform for them to come to learn, to be inspired, to grow’ (RC05).
	‘In our mind was people being isolated, lonely, and not knowing where to get support and we felt like we needed to respond to that’ (RC02).
Theme 5: changes to accessibility	‘It's one of those ‘you win some, you lose some’ kind of things’ (RC01).
	‘When I came in it was all online because of the pandemic and then we've tried to go back to face-to-face, which we have and we've got a hybrid model: some online, some not’ (RC25).

The term ‘workforce’ was defined as all individuals engaged in paid or unpaid full-time, part-time, sessional, casual or voluntary contracts at the recovery college. ‘Recovery college community’ was defined as all recovery college workforce and students. ‘Partners’ refer to any organisation or group external to the host organisation that was collaborating with the recovery college.

### Theme 1: complex organisational relationships

Recovery colleges were embedded in complex organisational relationships with partners, host organisations and local mental health services. Complex organisational relationships influenced changes to recovery college operation and their transition to digital delivery.

Some recovery college managers reported that their recovery college's position in relation to their local mental health services strengthened during the pandemic. Some recovery colleges were involved in the community mental health transformation project, a government initiative to integrate primary care networks with secondary mental health services and local third-sector organisations to reduce gaps in mental health provision. Recovery colleges are often accessible services, with no inclusion criteria or referral needed. As such, some recovery colleges were relied upon to relieve pressure on local mental health services that became inundated during the pandemic. Subsequently, recovery college curricula were sometimes tailored to focus more on mental health and self-management topics, where non-mental health courses were already being offered by other local organisations (e.g. the local education sector or third-sector community groups). Recovery colleges were subsequently considered an accessible source of support that could meet the needs of those waiting for local mental health services, potentially preventing individuals from requiring these services altogether.

‘We have definitely acted as a preventative service through the pandemic. We have been that first point of call to stop people going back into services or to stop people using services in the first instance’ (RC28).

Recovery colleges often had limited resources despite their importance in relieving pressure on local mental health services. Reciprocal relationships with partners were seen as essential to the operation of recovery colleges with limited resources and aspirations to deliver a wide variety of courses. Financial resources and equipment were exchanged alongside ideas for courses and community initiatives. However, organisational disruption caused by the COVID-19 pandemic left lasting challenges to sustaining effective partnerships.

‘People were kind of put on furlough [ … ] so, the opportunity to develop the community relationships was something that was massively, massively diminished’ (RC15).

Recovery college autonomy from host organisations also influenced recovery college operation during the pandemic. Generally, managers at third-sector recovery colleges described the transition to digital delivery as efficient and agile, whereas NHS recovery college managers reported a longer transition with less decision-making autonomy. For example, some NHS recovery college managers were instructed to use inaccessible videoconferencing platforms that adhered to NHS Trust information governance policies, but were inappropriate for group course delivery. Although some managers negotiated permissions to use accessible platforms, others cancelled courses in response to accessibility issues.

The responsibility to support mental health services during the pandemic was seen as both a success and a burden, as managers felt that recovery colleges were expected to deliver beyond their capacity.

‘They expect us to be able to do X, Y and Z. You kinda go, hang on, [ … ] we don't have tons of staff’ (RC18).

### Theme 2: changed ways of working

Organisational practices changed to overcome the disruption caused by COVID-19 to recovery college operation.

Face-to-face courses were initially suspended in March 2020, and courses delivered during the pandemic were sometimes cancelled or postponed because of facilitator and/or student sickness. It was not feasible to reliably reinstate socially distanced face-to-face course delivery at some recovery colleges, because of changing government-mandated lockdowns and difficulty accessing venues that could accommodate socially distanced course delivery.

‘One room we had designated to ourselves, [ … ] we could have five people in that room, but by [the] time you've got two of us in the College, it's not really worth running just for three people’ (RC29).

Managers hoping to return to face-to-face course delivery expressed future aspirations to acquire more accessible physical bases with large outdoor spaces in community settings over smaller office spaces. Managers who reinstated face-to-face courses had to reduce student attendance as a result of social distancing restrictions. Managers who did not wish to reinstate face-to-face courses closed their physical buildings, saving costs and improving workforce efficiency through home working.

‘In the middle of COVID as we are, we haven't got the [student] numbers that I needed that building space for’ (RC07).

Financial resources were also re-directed to accommodate reduced outgoings resulting from home working and termination of ongoing projects curtailed by the pandemic. New funding opportunities became available to facilitate pandemic-related priorities, such as transitioning to digital course delivery.

Maintaining and building a stable core team during lockdowns could feel challenging. Changes to roles, responsibilities and job security resulting from redeployment, redundancies, furlough schemes and team mergers spelt uncertainty for recovery college workforces. Managers felt that the workplace and pandemic-related uncertainties were burdensome on the emotional well-being of recovery college workforces, many of whom experienced pre-existing mental health challenges.

‘Because we have staff with lived experience, the impact of the actual pandemic on their own well-being [ … ] has been massive in some cases’ (RC21).

Informal online/telephone meetings became routine organisational practice to support workforce well-being and team identity.

### Theme 3: navigating the rapid transition to digital delivery

The transition to digital delivery was considered a key pandemic-related change to recovery college operation. Rapid transition from face-to-face to digital course delivery required new equipment and digital skills training for the workforce. Obtaining these resources was a challenge for recovery colleges with limited budgets, causing some recovery colleges to close temporarily. Networking with other recovery college managers and digitally competent workforce members assisted the transition to digital delivery. Improved cultural acceptance and access to means of online communication made enacting plans for digital course delivery easier to justify during the pandemic.

‘The time was right during COVID to create the online platform’ (RC04).

Reluctance to deliver courses online, NHS redeployment, staff restructuring and pandemic-related mental well-being struggles were common challenges to workforce retention at the beginning of the transition. For some recovery colleges, this resulted in there being fewer lived experience and topic experts available to participate in coproduction. Some recovery colleges offered a reduced selection of courses because of diminished coproduction. Managers with workforce members employed to create and maintain the coproduction processes experienced fewer challenges to continuing coproduction online.

‘We could develop these [ … ] courses that we think would be necessary and that did come [ … ] from feedback [ … ] so it was [ … ] very much based on the need highlighted [ … ] but actually the student involvement in the development of those courses was [ … ] greatly diminished’ (RC15).

Technological problems such as delayed audio meant students would unintentionally interrupt each other, deterring them from making further contributions. These barriers to student engagement in online courses left facilitators feeling uncomfortable sharing sensitive lived experiences, making facilitation feel unfulfilling in some instances.

‘Every single person on the class had their camera off and [ … ] I've told my story in a room like that and it's like speaking into the void. It's horrible’ (RC22).

Online breakout rooms were used to create smaller ‘study groups’, making facilitation and in-course discussions more conversational. Course facilitators trialled videoconferencing platforms to improve confidence in their use and build an appreciation of the platform's accessibility. Roles were created to provide support for students experiencing technical difficulties so that facilitators could continue uninterrupted, and online courses were shortened to reduce fatigue.

Methods to support student safety and confidentiality online were created, such as instructions on how to create a confidential space when participating in courses remotely. Individuals who became distressed during a call would often receive a private telephone or video call from a member of the recovery college workforce to debrief. However, managers felt it was difficult to create a sensitive and comforting environment to address safeguarding concerns and student distress virtually.

‘It's really hard to sort of talk to someone really, openly and comfort them when it's not face-to-face’ (RC20).

### Theme 4: responding to isolation

Members of the recovery college community missed the welcoming in-person recovery college environment, the spontaneous conversation and sense of community that came with face-to-face course delivery. Some managers felt that high attrition in online course engagement was related to challenges in creating a digital space that facilitated human connection.

‘There is something [ … ] very magical that happens in the classroom, face-to-face [ … ] you witness this amazing communication where students begin to answer their own questions and begin to help each other [ … ] we had many discussions around “would we get this ever again over running virtual courses?”’ (RC03).

As reinstating face-to-face course delivery was challenging because of COVID-19-related anxieties, a variety of means to keep students connected to the recovery college community while adhering to social distancing restrictions were used. Recovery college newsletters informing students about local activities and methods of coping during the lockdown were distributed.

Receiving digital skills training from the recovery college also generated tangible changes to students’ lives and recovery; enabling students to connect with their friends, families and wider social networks, as well as building their own sense of autonomy and confidence.

‘It didn't matter what the content of the course was it was more important that we show them that they were capable of going online and speaking to other humans and that opened a whole new world to them’ (RC08).

### Theme 5: changes to accessibility

Managers felt a duty to be inclusive because prospective students were likely to have unmet needs as a result of difficulty accessing inundated mental health services during the pandemic.

‘A lot of our clients get left behind from [ … ] families, from friends, from other support that they should be getting [ … ] So we had to make sure that we weren't included in that’ (RC05).

Various means of accessing and engaging with the recovery college were created. Recovery college websites and social media were updated with events and accessible educational materials such as self-help guides, podcasts and webinars. Students were consulted on ideas for developing pilot activities, courses and enrolment processes through feedback forms and/or focus groups. Offering a variety of courses that used a blend of face-to-face and digital delivery modalities was a common method of accommodating access preferences. Many managers expressed aspirations to maintain their hybrid offer post-lockdown.

Transitioning to digital delivery was felt to overcome physical access barriers for carers, people living in remote or rural areas, forensic recovery college students with restricted access to community settings, and people with physical and/or mental health difficulties who find face-to-face attendance challenging.

‘Recognising how many people in fact, that we had not reached because people could not attend our courses for whatever reasons. You know, either financial, mobility, [ … ] lack of public transport, anxiety around getting out of the house. But people could access courses online’ (RC04).

Managers identified that the demographic characteristics of students changed after the transition to digital delivery. Some recovery colleges enabled students nationally and internationally to attend their online courses. More men and younger people, but fewer individuals from marginalised ethnicities attended digitally delivered courses in some cases. More NHS staff joined online courses for their own mental health needs during the pandemic, fostering common connection with individuals they may otherwise categorise as patients in their professional lives. Course content and educational materials were also tailored to address the emotional well-being concerns experienced by NHS staff.

‘The impact that [ … ] the whole COVID situation would have on the NHS was very similar to the trauma that [ … ] armed forces have when they're out in any field of operation. [ … ] So we thought actually we can really apply some of our knowledge [ … ] and we built a self-help guide’ (RC08).

Digital poverty became a barrier to recovery college access during the pandemic. Online courses were challenging to access for members of the recovery college community who could not afford computer equipment or lacked digital skills. Resources to combat digital poverty and exclusion were acquired by recovery colleges with partnerships with technology organisations and access to funding. Evidence of coproduction in digital inclusion strategies at recovery colleges could support funding applications for computer equipment.

‘We've got a development group, which is a coproduced group, but it's students and volunteers and they worked out how they thought digital inclusion could work and that's what sort of backed up our bid for funding in regard to getting the iPads’ (RC27).

## Discussion

Two key pandemic-related changes to recovery college operation are highlighted: the use of some recovery colleges as accessible preventative services that relieve pressure on clinical mental health services, and a transition to digital course delivery and aspirations to hybrid delivery that was underpinned by a commitment to accessibility and inclusivity for those with mental health needs.

Psychologists’ case-loads and waiting lists for mental health services increased during the pandemic,^18^ indicating the need for innovation in relieving pressure on these services. Some participants observed that attending recovery college courses could be sufficient for students to feel that they no longer need local mental health services. This finding is supported by a pre-experimental study that identified recovery colleges as effective strategies to support self-regulation after finding a significant reduction in recovery college student self-rated anxiety 3 months after completing an online recovery college course, compared with baseline.^19^ Collaborations between recovery colleges and mental health services may be facilitated by the NHS Community Mental Health Framework,^20^ which advocates for improved access to community mental health services. However, recovery colleges are not replacements for clinical or therapeutic services.^2^ Access to finances, staff and equipment vary across recovery colleges depending on the strength of their relationships with partner organisations. Recovery colleges capacity to relieve pressure from clinical and therapeutic services may differ on a case-by-case basis.

Recovery college workforces in England reacted to the March 2020 government-mandated lockdown by transitioning to digital delivery or finding other means to connect the recovery college community.^21^ Many participants expressed desire to continue providing courses digitally post-lockdown. The coproduced pedagogical orientation uniquely positions recovery colleges to develop and deliver digital skills training and educational resources to support the well-being of individuals with mental health needs. Participants observed such digital skills training enabled students to connect with their wider social networks. As recovery college staff became equipped to provide digital skills training, recovery colleges could continue to be a valuable resource for mental health services in tackling the impact of digital poverty on contemporary mental health service provision.^22,23^

Recovery colleges are also likely to affect greater recovery-oriented change at the service level if given opportunities to develop an alternative culture to their host organisation.^24^ A benefit of recovery colleges embedded within mental health organisations is that they have a relationship with statutory infrastructures, but the autonomy to act outside of them.^25^ Close relationships with host organisations and collaborations with partner organisations brought funding, topic and lived experience experts, and other resources such as the digital technology required for recovery colleges to continue their operation and provide digital skills training during the pandemic. Autonomy to act beyond statutory infrastructures, such as NHS bureaucracy, enabled recovery colleges to use digital technology that met a variety of access needs and supported maintenance of a workforce required to coproduce and co-facilitate courses. Effective collaborations may therefore require a balance between harnessing the strength and quality of the relationship between recovery colleges and their host organisations, and supporting the identity of recovery colleges as autonomous from, and complementary to, mental health services.

More healthcare staff were felt to have attended recovery college courses, potentially because of the high burnout and psychological distress experienced by NHS staff following the pandemic.^26^ Participants noted that this increased interactions between healthcare staff and students, which may balance power dynamics known to be detrimental to the well-being of those accessing mental health services.^27^

### Strengths and limitations

This is the first multi-site study exploring the effects of the COVID-19 pandemic on recovery colleges in the UK. Input to the design, data collection and analysis from LEAP co-researchers brought nuanced insights, supported reflexivity, and improved the applicability and accessibility of the findings. Participant perspectives may be biased toward managers with positive attitudes to their recovery college's transition to digital delivery, as interviews were only conducted with those who agreed to be interviewed via video call. Moreover, demographic data was not collected from managers, meaning that relationships between the participants’ characteristics and themes in the data cannot be concluded. Managers who started their role at the recovery college during the pandemic were unable to provide complete accounts of pandemic-related changes to the recovery college, but were able to provide details of more recent pandemic-related adaptations.

### Future directions for research

Two risks of delivering courses digitally were highlighted in the interviews: (a) increased difficulty in managing student distress, and (b) increased peer trainer discomfort in sharing personal stories about their lived experience to virtual audiences that are not explicitly engaged. Many managers expressed future aspirations to offer hybrid face-to-face and digital course delivery options, yet published information on overcoming harms to digital peer trainer work is sparse.

Digital poverty and exclusion were also barriers to recovery college's digital provision, particularly for individuals belonging to marginalised ethnicities. The Digital Poverty Alliance recognises the relationship between ethnic minorities and digital inequality as underresearched.^28^ Further research is therefore required to explore how digital recovery college courses can be delivered in a safe and accessible way.

## Supporting information

McPhilbin et al. supplementary material McPhilbin et al. supplementary material

## Data Availability

The data that support the findings of this study are available from the corresponding author, M.M., on reasonable request.
