# Supplementary material for: Investigating the impact of the COVID-19 pandemic on recovery colleges: multi-site qualitative study
Source: BJPsych Open. 2024 May 16;10(3):e113. doi: 10.1192/bjo.2024.70 (PMC11363083; doi:10.1192/bjo.2024.70)
Supplement: McPhilbin et al. supplementary material [file S205647242400070Xsup001.docx]

**Supplementary material**

S1: Topic guide

**RECOLLECT Topic Guide for qualitative interviews (WP1)**

**For Interviewer**

Content will be used for 2 papers:

1. **Paper 03: The national impact of COVID-19 on mental health recovery support from Recovery Colleges: qualitative study**

The aim of this study is to explore

- How the pandemic has affected Recovery Colleges (including what it was like prior to the pandemic)
- How Recovery Colleges have operated during the pandemic
- How changes made during the pandemic, may be taken forward (if at all)

1. **Paper 04: The organisational context and history of RCs in England**

The aim of this study is to explore

- How and why Recovery Colleges were set up
- Choices that were made in during early development
- Changes which have happened since early development of the Recovery Colleges
- Future directions of the Recovery College

**Four Areas to cover:**

1. History of the Recovery College (Paper 04)
2. Pre-pandemic operation (Paper 03, Paper 04)
3. Modifications due to the pandemic (Paper 03)
4. Post pandemic plans (Paper 03, Paper 04)

**TOPIC GUIDE BEGINS**

**Introduction**

Thank you for spending the time to talk with us today. Although we call this an ‘interview’, it’s much more of a conversation. We are looking to gain a deeper understanding of how Recovery Colleges operated in the past, how they have changed as a result of the pandemic, and the future direction of Recovery Colleges.

**We’re impartial and independent observers who are here to learn; this is not an evaluation. Every college is different so there are no right and wrong answers.**  Please feel free to say as much or as little as you like in response to each question.

Similarly, if there are any questions that you don’t feel comfortable answering then please do ask to skip the question. You don’t have to tell me why. Also, please feel free to pause the interview to take a break or stop the interview at any point.

Everything you say will be kept confidential unless you disclose to us any unreported criminal activity or that either you or someone else is at risk from harm, in which case we will have to inform someone else. This is because we need to do what we can to keep everyone safe. Any quotes we use in publications or outputs will also be anonymised.

I will begin the recording shortly. We will have to use video recording but please be assured that we will only be using the audio from the recording. Please do feel free to turn your camera off if this is more comfortable for you or if you don’t wish to be video recorded.

I will now start the recording and the transcription.

*START RECORDING

*[Note to interviewer: note all components identified as modified during pandemic on the survey and explore in Section 3 questions.]*

*[Note to interviewer: Do not ask managers for their full name at any point in the interview]*

**Opening Question:** Can you tell me about how you first got involved in the Recovery College?

*Prompts (if needed)*

- *What is your role?*
- *Is this the first Recovery College you have worked in?*

*[Note to interviewer: depending on the opening questions, some /all questions in Section 1 may be difficult to answer]*

**Section 1: History of the RC (Paper 4)**

1. Can you tell us when and why your Recovery College was set up?

*Prompts (if needed)*

- *Where did the idea come from?*
- *What was the Recovery College set up to achieve?*
- *How does the Recovery College fit with wider activities and services in the organisation?*

1. Could you tell us a briefly about the early development of the Recovery College?

*Prompts (if needed)*

- *Key people or groups involved in development*
- *Things that helped*
- *Key challenges or obstacles (e.g. administrative/bureaucratic, resistance, resources)*

Could you tell me about how the Recovery College has changed since it has been opened?

*Prompts (if needed)*

- *What were the key changes?*
- *What was the process of change?*

**Section 2: Pre-pandemic operation (Paper 3 and Paper 4)**

1. What are the most important components of your Recovery College?

*Prompts (if needed)*

- *[Check Q’s 22-31 (particular groups catered to) on survey responses] In the survey, you mention that your Recovery College caters to [group(s) of student], in what ways is this group catered to?*
- *What do you think is most important for the students?*
- *What do you think is most important to the staff?*

1. What does your Recovery College do to support students in working towards their goals?

*Prompts (if needed)*

- *Who or what influenced that decision and why?*
- *Who does this approach work better or worse for?*

1. Prior to the pandemic, what were the main barriers for students wanting to access your Recovery College?

*Prompts (if needed)*

- *Did the target student group(s) feel the college was relevant to them?*
- *Please could you tell me more about any access barriers you’re aware of at the Recovery College?*
- *Were there other options you were aware people used instead of your college? If so, why?*

**Section 3: Modifications due to the pandemic (Paper 03)**

1. How did you keep the Recovery College running during the pandemic?

*Prompts (if needed)*

- *How were the values of the Recovery College implemented during the pandemic?*
- *How were the workforce prepared to manage pandemic related changes at the Recovery College?*
- *If new technologies were implemented, how were students and staff supported in using this technology?*
- *Who or what helped implement these changes?*

1. How has the approach to co-production and adult education at the Recovery College changed since the start of the pandemic, if at all?

*Prompts (if needed)*

- *Have you noticed any changes to the relationships between staff and students?*
- *Have you noticed any changes to the process of co-production and adult education?*

1. Please tell us more about any other changes you’ve noticed about the Recovery College since the beginning of the pandemic.

*Prompts (if needed)*

- *[Check Q’s 60-72 (fidelity changes due to the pandemic) and Q’s 166-167 (budget changes due to the pandemic) on survey responses] You mentioned in the survey that XXX changed as a result of the pandemic, please could you tell us more about this?*
- *Any changes to how Recovery College managers communicate with each other?*
- *Any changes to how managerial decisions are made?*

1. Have you noticed any changes to equality, diversity, and inclusion at the Recovery College since the start of the pandemic?

*Prompts (if needed)*

- *Are there any areas that you think have improved?*
- *Are there any areas that you would like to see improve further?*

**Post pandemic plans (Paper 03, Paper 04)**

1. What changes that have been implemented at the Recovery College during the pandemic do you intend to keep, if any?

*Prompts (if needed)*

- *Who or what influenced that decision and why?*

1. What are the next priorities for the development of your Recovery College in the future?

*Prompts (if needed)*

- *How would you like to see the Recovery College develop in the next 2 years?*
- *What future changes would you like to make?*

1. Is there anything else you would like to add before I stop the recording?

Thank you for generously taking the time to talk to us and answer our questions. I will now stop the recording.

*STOP RECORDING

What we’ll do now is send the audio to be transcribed. We will then compare the transcript with transcripts of our interviews with other Recovery College managers to find patterns and themes. If you have any questions, please do let us know or email us at RECOLLECT@kcl.ac.uk .

**TOPIC GUIDE ENDS**

S2: Framework version 1

**
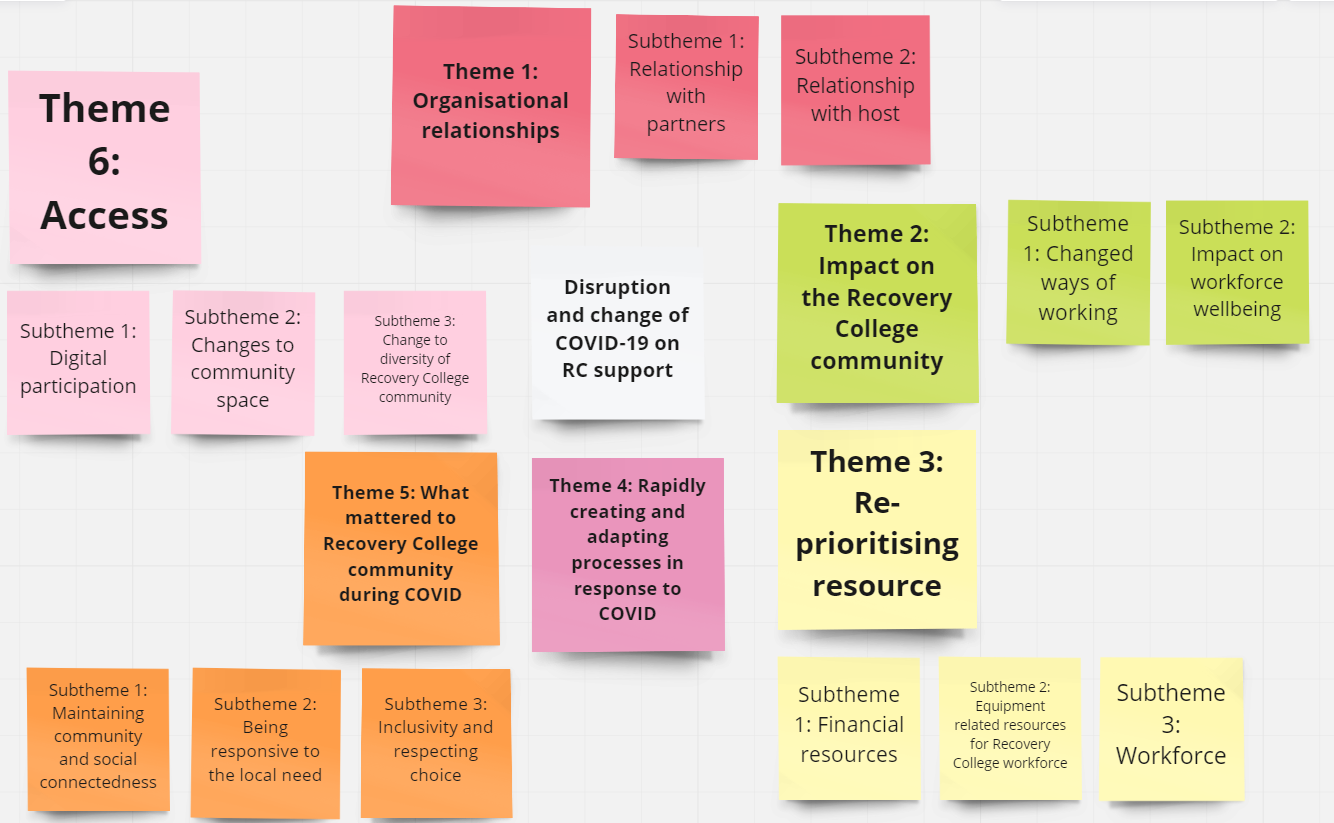
**

S3: Framework version 2

S4: Framework version 3

S5: Quote table

| **Theme** | ***Quote* [ID]** |
| --- | --- |
| ***Theme one: Complex organisational relationships*** | *So, I described our Recovery College in COVID as being a third-party sector provider in an NHS system* [RC02] |
|  | *Secondary care services are not still not doing an awful lot of face-to-face in-person stuff, which then means a kind of demand backs up. So, we're getting more asked of us* [RC18] |
|  | *There started to be this real goodwill emerging, there already was, but […] it was different before the pandemic. People weren't quite as willing to share like course materials and stuff like that* [RC21] |
|  | *Another positive is that we are integral now to our mental health system. Uh, and we've worked hard over the last 4, 5 years to go from an organisation, like a project of ‘is this going to stay? Is that gunna stick?’ to now being a central part to the delivery for our community mental health transformation, the training for our communities, our voluntary and community sector and our staff members too.* [RC17] |
|  | *It’s not just COVID that’s happened there’s the primary care networks that have happened and there’s an integrated care system that happened, there’s mental health transformation that’s happened. So, Recovery Colleges this year last two years probably faced alongside other people some really big challenges and to find it, find its place and position* [RC02] |
|  | *We get talking therapies who are very busy and have long waiting lists, signposting people to come to the college to access this course as a holding platform. So, we've got a big, we now have a waiting list but it's really important because quite often having attended the 11-weeks course people would decide they don't need the talking therapies so…. But, you know, their needs have been met and so, you know, they go off and they're quite happy.* [RC03] |
|  | *We've evolved depending on need because we're actually wanting support our CMHTs as well. So, what we then decided to do was to very much specialize in mental ill health strategies. So rather than be like a Recovery College that offers, uhm, art groups, music groups, those kind of groups, we, we felt that they were, could be offered in the community within the voluntary sector.* [RC30] |
|  | *We have definitely acted as a preventative service through the pandemic. We have been that first point of call to stop people going back into services or to stop people using services in the first instance.* [RC28] |
|  | *we get talking therapies who are very busy and have long waiting lists, signposting people to come to the college to access this course as a holding platform. So, we've got a big, we now have a waiting list but it's really important because quite often having attended the 11-weeks course people would decide they don't need the talking therapies* [RC03] |
|  | *We just give stuff to people and then if you give stuff to people, other organisations etc., they’re actually usually quite nice to you back.* [RC18] |
|  | *People were kind of put on furlough and bits and pieces like that. So, the opportunity to develop the community relationships was something that was massively, massively diminished, and also it is so vital for the success of what we do here* [RC15] |
|  | *Obviously, I mean there was a period of about three months when basically everything stopped for everyone and other people have resumed at slightly different rates. But from our point of view, the fact that we've got, you know, five floors and god knows how many thousands and thousands of square feet that it adds up to, I should know but… something like 20-30 thousand square feet or whatever it is…Um has meant that, you know, we’ve been able to kind of foster more collaboration. We’ve just said to people, “Look, we’ve got big open spaces, bloody use them.” And, you know, but [long pause]. Yeah, I still don't think anybody is operating really at full tilt.* [RC18] |
|  | *We're very lucky here in that we're part of a Trust. We're not, we're not commissioned. So, we're just funded by the Trust. Umm. So that gives us a certain amount of kind of freedom, you know, to in terms of how we kept going.* [RC25] |
|  | *A couple of the voluntary and community sector partners were quicker getting on to Zoom and delivering. They're not governed by the same tight NHS regulations that we had so they, some were quicker than ours.* [RC17] |
|  | *We were hit with this that the Trust, wouldn't buy license for our service or anywhere. So, Microsoft Teams is what we've told we've just got to use, which doesn't, huge issues with it but we keep trying and we're working very hard with our information governance and IT department at the moment. You know they've looked at our technology, but the biggest problems come because of the technology that student, Microsoft team takes a lot more bandwidth, the audio control, everything like that is not good enough for our students that a lot of them were just using phones or iPads, or they're in a home with very poor Wi-Fi connection. So huge issues with services and we've had to this term, we’ve had to cancel a few workshops. We didn't feel it was fair to put students through a couple. We had disastrous couple of sessions, people almost in tears, trainers who were struggling because it, people get getting cut off and but anyhow, that's so where we are at the moment.* [RC29] |
|  | *Sometimes other people think we're more cap-, not more capable than we are, think that we think that we’ve got almost more capacity than we have. And so, they expect us to be able to do X Y and Z. You kinda go, hang on, you know we don't have tons of staff. You know? You know, people get into, “Oh can your PA not sort that out?” No, don't have a PA. Who the hell’s got a PA? You know, you know. [Laughs] We do our own scheduling, we do our own emails, do our own, yeah. So that's, um, I dunno, a drawback of having a degree of success is that people almost kind of see you as being the solution to their problems, and it's like, well, could you ask us first? [Laughs] You know, if we can do that. So that's a bit awkward.* [RC18] |
| ***Theme two: Changed ways of working*** | *It just feels it feels quite relentless through the constant change, constant adapting* [RC19] |
|  | *Our provision like I say it has completely changed* [RC27] |
|  | *Because things have been so up and down. It's been like or we can open now closed and that sort of thing we've just done our prospectus over a couple of terms. So, it's been difficult sometimes to plan things* [RC26] |
|  | *We did put a stop to face-to-face sessions, naturally, as we had to follow national guidelines.* [RC15] |
|  | *Staff sickness, staff isolating. At times, you know, when the wards were shut, we weren't able to facilitate* [RC06] |
|  | *I think we were possibly the only Recovery College that went back to in person in the autumn 2020, a little brave-stroke-foolish. I think most people stayed online, but we wanted to do both, so we went back to in person as well. We had to then cancel everything in [month] when we went back into lockdown, but we did have in person again for [a year later] and then we had to cancel everything in person, and we did [a few months ago] purely online.* [RC14] |
|  | *One room we had designated to ourselves, although others would use it on the days that we weren't operating, is small with no window, very small and we've deemed it no, not, well, we could have five people in that room, but by time you’ve got two of us in the college, it's not really worth running just for three people. So yeah, we're struggling at the moment to find venues across, that aren't already booked* [RC29] |
|  | *I thought the only sensible thing here is to close the building actually because it doesn't give me any flexibility, and at the moment just post-COVID or kind of in the middle of COVID as we are, we haven't got the numbers that I needed that building space for* [RC07] |
|  | *One of those things that is a concern is attendance to those sessions because we have to limit numbers, which mean if a couple people don’t come and you’ve got a waiting list, of people wanting to come, it irks a little bit* [RC02] |
|  | *I think that has made a real difference. Actually, having some virtual capacity. It is easier for staff and there's something about, I don't know if it was something about yeah staff getting time to go to a location to do a face to face course or whether there was more like a oh gosh, is there one on my caseload going be there* [RC09] |
|  | *I think that has made a real difference. Actually, having some virtual capacity. It is easier for staff and there's something about, I don't know if it was something about yeah staff getting time to go to a location to do a face to face course or whether there was more like a oh gosh, is there one on my caseload going be there* [RC09] |
|  | *'Cause, we were entirely online and over the telephone we were running I reckon maybe like maybe four times the amount of groups that we were doing compared with when I started when we were face-to-face. So actually, all provision and on our offer increased a hell of a lot.* [RC15] |
|  | *We just kind of aligned to ok, well what money do we have in the coffers and, you know, what money is coming in and 'cause we've done our budgets for face-to-face delivery but you know everything from travel, accommodation, you know all of that, we all under spent massively on our budgets because we weren't sort of going out and that out and about doing activities so everything online was cheaper to do so we just cut our cloth accordingly* [RC08] |
|  | *Through pandemic funding during the lottery, [name of charity] won a contract won [thousands of pounds] or something like that and decided to fund an extra post that's half time with us, and half time with [name of charity] and his role at [name of charity] is to set up recovery groups online.* [RC14] |
|  | *We did obviously have to take massive advantage of the furlough scheme. We have done a complete restructure and you know lots of people were made redundant.* [RC08] |
|  | *I think the biggest one was actually the merging. The merging of the two teams particularly in developing new infrastructure around that* [RC15] |
|  | *Because we have staff with lived experience, the impact of the actual pandemic on their own wellbeing and their own fears and worries and anxieties has been massive in some cases.* [RC21] |
|  | *We set up pairings in the team and they changed every week (clears throat) and you had to have a telephone call, not an onscreen telephone call with your buddy at some point that week, just as a sociable- what you would have had while you were waiting for the kettle to boil or when you walked over the road to get a sandwich, those kind of conversations that we suddenly weren't having. Uhm, and that didn't always go down very well, people find it very forced and- but it also, for others, was very welcome, just have some social contact.* [RC24] |
|  | *We did use to try and get people together for like a weekly chat really with all the volunteers and stuff to try and keep people going and in the end set up a buddy system but one of them came up with a quote of something, it's something along the lines of “we're all in the same storm, but we're not all in the same boat”, and they all kind of realized that then, that meant you know some people are going to want to do this, some people need to, someone may not be able to.* [RC28] |
| ***Theme three: Navigating the rapid transition to digital delivery*** | *We had to survive. We had to offer people something and we had to, we’re very, we were very aware that our students were left with nothing* [RC03] |
|  | *It had to be kind of quite reactive and quite responsive at the time, we didn't really have that opportunity to set up official processes for it because, you know, actually the focus was on getting people online and engaged to some degree as quickly as possible* [RC15] |
|  | *We wasn't set up to deliver online, we wasn't set up to work remotely. You know, so we actually had to go out and buy quite a lot of IT equipment to make sure that we could offer that* [RC15] |
|  | *I guess another factor that was challenging is that we have such a small budget, and we were expected to buy all new laptops. We had volunteers that didn't necessarily have computers at home, so we had to try and get laptops out to them and that that was difficult. I would say the actual laying our hands on physical equipment was probably more of a challenge than actually getting used to the software.* [RC20] |
|  | *I run daily CPD hints and tips of just normal, you know, how do you change your screen on Teams or something because in the office you would suddenly, you'd see someone doing something it be like. “How did you do that? How did your screen just split in two”, you know, and you really quickly show somebody something new and they go, “Oh wow, you know that's completely changed my way of working.” We don't have that anymore actually and some, you know, different team members are really, either sort of really agile at learning new things and sort of saying “hey, I've just noticed you know, how did you appear twice on the screen” (laughs) and they're good at asking and other people just sit quietly thinking “I'll just carry on doing what I've always done.* [RC08] |
|  | *We were quite lucky. Our coordinator was quite tech savvy and definitely lead on things like our podcasts and things like that.* [RC16] |
|  | *We were part of the ImROC Recovery College, sort of, what are they called? Learning sets at the time. So, we went from doing those face to face to virtual. So that was, what was quite useful was they started doing lots of emails, people were emailing back and forth going ‘we've done this’, or sharing, like, things like uhm, ‘we have set up a webinar’, or like, you know, the kind of guidance on them and things like that. So, there was lots of sharing of bits. That was quite helpful* [RC16] |
|  | *We had thought about going and offering online courses, but I just felt that it wouldn't have been the time. I don't think people would have really, it wouldn't have had the momentum or the, I don't know, just the time was right during COVID to create the online platform* [RC04] |
|  | *We've always wanted to go online but actually we didn't really know how and what that would look like and the IG hoops that we would need to jump through for being an NHS service, you know, it’s incredible but it forced the issue and it's proved that it works.* [RC17] |
|  | *We found a lot of people now have that means that wouldn't have had it completely years ago.* [RC13] |
|  | *We found it really difficult to engage people in co-production online. It just [shakes head]. Yeah, we did try, but at the same time I think our efforts, not through the want of trying, but it was circumstantial as well. Actually, we couldn't operate in the same way, you know, meeting students and supporting students when we're face-to-face. We can have that recap after the group, we can have that supervision after the session. Offering that same level of support over the telephone or over Zoom. It was really, really difficult. We did have some groups continue to be co-facilitated, but they massively reduced down in numbers, which was an unfortunate by-product of it and the same for the development of courses. It got- it was a conscious decision. We could have carried on with the six courses that we had, the six or seven courses that we had set up which had been co-produced. It would be very difficult to deliver more if we have relied on that co-production. So, we made the conscious decision well actually, we could develop this kind of variety of courses that we think would be necessary and that did come-, some of that did come from feedback. we do kind of student satisfaction surveys and bits and pieces like that where people could have that input. So, it was kind of very much based on the need highlighted and the need highlighted in the initial assessments, but actually the student involvement in the development of those courses was kind of greatly diminished.* [RC15] |
|  | *We went through quite difficult time in that people that we’ve have normally sort of come in with co-production with us, some of our volunteers and our experts by experience. We had to fight really hard to maintain those relationships. People were nervous people were scared at home and I think people, like with the media response and everything else in it definitely had an impact on people's mental health. So, we had some of our experts who were just not in a place to sort of work with us at that time.* [RC20] |
|  | *When service users are used to being around a big table and you know we'll meet on Tuesday morning, have a cup of coffee and you know and you know it, it, it's a, it's a very difficult different kind of and, and yeah, there, there were technical issues and some people just don't like this. It’s not, not because they can't do that. They just don't like it and don't want to do it. Thank you very much.* [RC19] |
|  | *We've got focus groups all over the place for particular courses, and students are really ingrained in that development and that delivery of the service. Well, sorry, ingrained is probably the wrong word, we're starting to get that back again to where it where it once was and it's a key factor of the service and we want that co-production and we've actually got a co-production lead within the service who's responsible for ensuring that that process is followed appropriately. So, when we do have an idea for a new group, when a member suggests a new group, it actually goes through all of the right stages and especially when a when a wonderful position where we have got so much content, there's no mad panic. There's no immediate need to have new sessions introduced so we can do our due diligence with that and make sure that the students do have the involvement they should have and we can get those peer facilitators in place as well.* [RC15] |
|  | *Every single person on the class had their camera off and I've done that and I've told my story in a room like that and it's like speaking into the void. It's horrible. You know, you don't even know if they're there. It's awful but then again they don't want to be on camera.* [RC22] |
|  | *We did have a couple of people who had been linking in with the team under the sessional rate payments, who very quickly said “I love working with you guys, but this is not for me. I do not want to see myself on a screen. This is like literally my worst nightmare. I do not understand it. I do not get it. Let me know when you are doing face to face stuff in [county] and I'll come and help out again”* [RC09] |
|  | *Another barrier was it had a time lag, so presenters would be talking over each other at times and students were trying to ask a question and think they'd managed to grab a silence and actually they, it came in on- so, we actually found interaction dropped because people were just afraid of interrupting and talking over somebody else, so that was harder.* [RC24] |
|  | *We've also done things like, sort of, study groups 'cause what we were missing in a delivery, it feels a bit more like you're delivering too people when you're online and you haven't got the time space* [RC16] |
|  | *For the rest of the team that time to build confidence and just to play around with all the functions and it doesn't matter if it goes wrong* [RC09] |
|  | *We did some research and Zoom came back as being more user friendly. So, we asked our learning and development team if they had any views on that and they said that for people with additional learning needs that Zoom tends to be a bit more straightforward and that aided our decision to go with that.* [RC20] |
|  | *We have a host who's our tech bod who sits behind the scenes and if anybody’s struggling to get on or is having any IT issues, they try and support them while the trainers are still, kind of, able to deliver.* [RC16] |
|  | *Most of the members of the team, the majority of the team said actually, an hour maximum feels like long enough. So, compared to our face-to-face courses where we'd meet, you know, for two hour session with a break in between, we're like, ok, we won't do whole courses. We will do virtual workshops, and they're going to be a maximum of an hour long.* [RC09] |
|  | *In the classroom we usually do you know that sort of class agreement and how we work together, that’s not so prescribed in the classroom, on Zoom it was fairly prescribed and long, we had three pages because we had to educate people about how the tech works. And cautions and things, like make sure you're not sitting in front of your phone bill magnet up on the fridge, you know that kind of thing* [RC22] |
|  | *If you just a bit worried about someone it's really hard to sort of talk to someone really, openly and comfort them when it's not face-to-face.* [RC20] |
|  | *In a classroom setting, you've got all these abilities to, kind of like, if somebody’s struggling with something or you need a little bit of time, you can, kind of, set a little bit of individual or group work and then you can quietly talk to somebody. When you’re online, everybody sees the one person you're talking to* [RC16] |
|  | *Face-to-face when they walk in, you know, some people who have been self-neglecting and all of this you, you get those clues which online, is harder to do.* [RC01] |
| ***Theme four: Responding to isolation*** | *Our clients, they needed a space where they were meeting people. You know, a lot of them are vulnerable, they were confined to their rooms wherever they were living, and we needed to have a platform for them to come to learn, to be inspired, to grow despite that they were, you know, had to stay in one place.* [RC05] |
|  | *In our mind was people being isolated, lonely, and not knowing where to get support and we felt like we needed to respond to that* [RC02] |
|  | *We've stopped having the centres, a lot of [RC population demographic] are really unhappy about the fact that they no longer have a place that they can just turn up and go and have a coffee and chat to someone friendly* [RC08] |
|  | *There is something sometimes quite often very magical that happens in the classroom, face-to-face when as a trainer, whether you're a peer trainer or practitioner trainer, you sit back and you witness this amazing communication where students begin to answer their own questions and begin to help each other. And I go tingly, as I'm saying that 'cause I, it's happened so many times, but it never fails to have such an impact on me? And we were, we were worried and we had many discussions around ‘would we get this ever again over running virtual courses?’* [RC03] |
|  | *We've had caught a substantial decline in numbers since the pandemic.* [RC19] |
|  | *I think with virtual because yeah there are, there's definitely the engagement in attrition levels and commitment for students going, you know, because it is a bit easier to tap in and out.* [RC09] |
|  | *And what we're seeing a lot of now as we're running more face-to-face sessions is people who are still really anxious about COVID, you know, we keep hearing like things from a handful of students where they haven't really left the house in two years, and so the idea of doing that just feels monumental.* [RC31] |
|  | *This last term, the [name of the month of the last term] term, our face-to-face attendance was really low and we cancelled quite a lot of courses, so that was money lost. This term our face-to-face, currently people booked in is higher. So, we're hoping that as, you know, anxiety reduces our courses will get fuller again.* [RC24] |
|  | *that was really important to our patients. From the start, they said they didn't want to lose that, sort of, contact with each other* [RC06] |
|  | *I think we did try really hard to offer as many different ways of connecting with people as we could in the pandemic* [RC14] |
|  | *So especially at the start of the pandemic about all the food banks, all the care packages, where to pick them up and which CCG was offering, you know, which well-being kits and all of those things. So we then started create- we would collate all that information and send it out to people.* [RC01] |
|  | *one of our students we literally had to hand hold him all, every step into the course because he was like “I don't even know how to email, I don't know how to, you know there's no way I can do this.” And after the meeting greet, they literally, the feedback to their case manager was like” my whole world has changed. I can now you know, Zoom with my family because I know how to do it” or “I can, you know, I can now speak to friends online. I'm no longer alone” and it was like God, you know that you know it didn't matter what the content of the course was it was more important that we show them that they were capable of going online and speaking to other humans and that opened a whole new world to them* [RC08] |
| ***Theme five: Changes to accessibility*** | *It's one of those ‘you win some, you lose some’ kind of things* [RC01] |
|  | *When I came in it was all online because of the pandemic and then we've tried to go back to face-to-face, which we have and we've got a hybrid model: some online, some not* [RC25] |
|  | *A lot of our clients get left behind from, you know, from families, from friends, from other support that they should be getting, maybe it's off the government or whatever, you know. So we had to make sure that we weren't included in that.* [RC05] |
|  | *So we are, we have a week every term where we gather feedback from our clients and that's actually embedded into the courses that we do and it's- uhm, I mean, we ask the clients loads of different questions about how the, you know, the logistics of how the courses are run, the content and lots of other things.* [RC05] |
|  | *Not only did we develop the existing content to be able to deliver online; there was a huge, huge push to develop new content as well.* [RC15] |
|  | *I think largely our referral systems used to be kind of contacting the office, it used to be telephoning the office. Then someone would upload details onto our database and then someone would contact them or speak to them there and then it was a little bit convoluted. It wasn't quite as straightforward as it can be. However, shortly after I started, and this isn't anything to do with me I'm not blowing my own trumpet, it was already in place by my head of operations. There's an online web form now that you can just go onto our website, you can do it, another professional can do it, your mum can do it, anyone can do it. It's completely open as long as the person knows that the referral is actually happening, they can just log onto our website, complete the registration form, and then they're added to our added to our waiting list for the service* [RC15] |
|  | *It's always a challenge when you have again, because you have such a diverse group of people, you know, some people are really used to it, some are not. Trying to, sort of, accommodate everyone's needs can be quite tricky sometimes.* [RC06] |
|  | *For the last two terms, we've offered some outdoor face-to-dace activity because we do recognize we do have a cohort of students that are still really missing that face-to-face activity. A lot of people have access digitally. UM, some have chosen not to, and have just said no* [RC27] |
|  | *Recognizing how many people in fact, that we had not reached because people could not attend our courses for whatever reasons. You know, either financial, mobility, transport, lack of public transport, anxiety around getting out of the house. But people could access courses online.* [RC04] |
|  | *We've got a new cohort of students that said, we would never have come, people that have got physical disabilities, people that have got caring responsibilities or you know may have young children, that said, we just couldn't access before.* [RC27] |
|  | *There are meetings and forums which we used to attend in the community where it was really hard to get some of our patients to. Because obviously we have to, you know, think it's a forensic site, we have to think about patients leave, uhm, sort of, areas they can go to. So, you know, we had some consultations with Rethink recently and we were able to bring so many patients to that we were reviewing, uhm, the Mental Health Act. So yeah, I mean, it's just, it's definitely, you know, initially it felt like a barrier, but now it feels like having this opportunity to offer things on Teams opens up so many doors.* [RC06] |
|  | *The other thing about with the Recovery Colleges going online, it's we've had international representation from Canada, USA, Bosnia, Finland. So, we've had that real interest coming in and it's joined the UK. So, the current Recovery College, [acronym for name of lived experience recovery group], which is a [name of lived experience recovery group] in Scotland, England and Wales at the moment. So, we've got that that real unity across the UK* [RC13] |
|  | *We've run digital drop-in groups every week at all tests where people can come in and learn how to use devices but it still had a massive impact on engagement from the BME community has fallen considerably.* [RC21] |
|  | *We've worked with our staff, not many of them, you know, we're at the very beginning of this journey but what we notice it initially was we had more staff attending our courses online. So where would all be external emails, we during the pandemic earlier, well probably the beginning of this year we noticed a steady increase in the number of [name of NHS Foundation NHS Trust] staff emails that were registering for our workshops. And so, we started to report that, we started to say “look staff are coming to this”, and they were coming to things like kindness, gratitude and some of our cultural and arts courses and a number of them actually were coming to courses around understanding and managing things like depression, anxiety, and the feedback we were getting on courses was really interesting. So, we had some of our clinical staff say: “we're here because we know everything about depression, but actually, having done this today, we've realized we don't, that we need to hear about it from the users perspective.”* [RC10] |
|  | *The impact that this situation, that the whole COVID situation would have on the NHS was very similar to the trauma that you know, armed forces have when they're out in any field of operation. So we thought actually we can really apply some of our knowledge.* *Some of our learning and we built a self-help guide* [RC08] |
|  | *It got raised about all staff must be really struggling. So, we decided to run a couple of retreats basically that are aimed at health care staff.* [RC11] |
|  | *We're very, very aware of digital exclusion. So, we know that there are a bunch of students out there that haven't been able to access our virtual courses due to, uhm, due to skill set, due to not having the right equipment or having the equipment but not knowing how to use it* [RC03] |
|  | *I think right across the board we may have got between about 30 or 40 tablets, purchased for our students so we could give those out to members who couldn't access.* [RC15] |
|  | *there was nothing that we could really do within those time scales to make sure that people were able to access the tech that was required but the only thing, so we obviously couldn't like, you know, sort of send smartphones and laptops to people. That wasn't something that was within our, you know, capability and capacity* [RC09] |
|  | *we're in the process of creating a comprehensive digital starter pack and this is going to be great 'cause it basically would be a set of physical resources that they can use, video, pre-recorded videos that are made that staff can watch, like train the trainer sessions that staff can watch and lesson plans and this will all, this will help the staff teach pre-entry level digital skills to clients. So how is set up a smartphone and use the functionalities of a smartphone, how to set up a tablet or a laptop and then use the main functionality of that up to the point where they can get onto a virtual session and then they can join the virtual, they can join the digital college* [RC05] |
|  | *We've got a development group, which is a co-produced group, but it's students and volunteers and they worked out how they thought digital inclusion could work and that's what sort of backed up our bid for funding in regard to getting the iPads*. [RC27] |
